# Allogeneic hematopoietic cell transplantation for patients with *TP53* mutant or deleted chronic lymphocytic leukemia: Results of a prospective observational study

**DOI:** 10.1038/s41409-020-01013-y

**Published:** 2020-08-16

**Authors:** Johannes Schetelig, Jennifer Hoek, Stephan Stilgenbauer, Jan Moritz Middeke, Niels Smedegaard Andersen, Christopher P. Fox, Stig Lenhoff, Liisa Volin, Avichai Shimoni, Wilfried Schroyens, Michel van Gelder, Donald Bunjes, Anja van Biezen, Henning Baldauf, Liesbeth C. de Wreede, Olivier Tournilhac, Nicolaus Kröger, Ibrahim Yakoub-Agha, Peter Dreger

**Affiliations:** 1 University Hospital, Dresden, Germany; 2 DKMS Clinical Trials Office, Dresden, Germany; 3 European Society for Blood and Marrow Transplantation (EBMT), Leiden, Netherlands; 4 European Research Initiative on Chronic Lymphocytic Leukemia (ERIC), Barcelona, Spain; 5 EBMT Data Office, Leiden, The Netherlands; 6 Ulm University, Ulm, Germany; 7 Rigshospitalet, Copenhagen, Denmark; 8 Nottingham University Hospitals NHS Trust, Nottingham, UK; 9 University Hospital Lund, Lund, Sweden; 10 Helsinki University Central Hospital, Helsinki, Finland; 11 Chaim Sheba Medical Center, Tel-Hashomer, Israel; 12 Antwerp University Hospital, Edegem, Belgium; 13 University Medical Center Maastricht, Maastricht, The Netherlands; 14 Medical Statistics, Department of Biomedical Data Sciences, Leiden University Medical Center, Leiden, The Netherlands; 15 Service Therapie Cellulaire & Hematologie Clinique, Centre Hospitalier Universitaire, Clermont Ferrand, France; 16 University Hamburg Eppendorf, Hamburg, Germany; 17 Centre Hospitalier Universitaire de Lille, LIRIS, INSERM U995, Université de Lille, Lille, France; 18 Department of Medicine V, University of Heidelberg, Heidelberg, Germany

**Keywords:** Chronic lymphocytic leukaemia, Stem-cell therapies

## To the Editor,

Patients with chronic lymphocytic leukemia (CLL) with a deleted or mutated *TP53* gene (*TP53*_mut/del_) have the worst prognosis among genetically defined CLL subgroups. Disruption of TP53 function confers resistance to chemotherapy and leads to less sustained disease control on pathway inhibitor treatment [1, 2]. In contrast, in several studies of patients who received alloHCT, presence of *TP53*_mut/del_ was no significant risk factor [3–7]. Progression-free 5-year survival rates after alloHCT ranged between 33% and 45% and many long-term survivors were MRD-negative [5, 6, 8]. In order to evaluate alloHCT for patients with *TP53*_mut/del_ CLL in first or second remission prior to transplantation, a prospective observational study enrolled patients between 2010 and 2012. Here, we report long-term outcomes.

The study was conducted as a registry-based prospective observational study by the Chronic Malignancies Working Party of EBMT. Eligibility criteria were as follows: Patients had to be scheduled to receive HLA-compatible related or unrelated alloHCT for 17p-/*TP53*-mutated CLL. Patients had to be in first or second complete (CR) or partial remission (PR) at the time of alloHCT. Patients with >1 mismatch at HLA-A, HLA-B, HLA-C, or HLA-DRB1, ≥70 years of age, ex vivo T-cell depletion of the graft or intended in vivo T-cell depletion with alemtuzumab were excluded. All patients signed informed consent. The trial complied with the declaration of Helsinki and was registered with clinicaltrials.gov (NCT01675102).

Patients who achieved a CR were tested for minimal residual disease (MRD) in peripheral blood and bone marrow. Testing for MRD was done locally using flow cytometry or molecular analyses. The primary endpoint of the study was to analyse relapse-free survival (RFS) at one year after alloHCT with a null hypothesis of ≤50% RFS at that time point. Details on data management and the statistical analysis are provided in the supplement. Data were analyzed as of August 31, 2019.

A total of 41 patients were enrolled at 11 centres between June 2010 and September 2012. One patient with Richter’s Syndrome and one patient aged >70 years at alloHCT did not meet the eligibility criteria, and were excluded from the study after review of the data. The final analysis set therefore included data from 39 patients, 28 male and 11 female patients. Median age was 59 years (range, 28–69 years). All patients had a Karnofsky Performance Score ≥80%. The median hematopoietic cell transplantation-comorbidity index (HCT-CI) was 0 with a range from 0 to 9. Thirty-five patients (90%) had a deletion 17p confirmed by FISH with or without a *TP53*-mutation. Four patients (10%) had a *TP53*-mutation only. The deletion (17p)/*TP53*-mutation was diagnosed during the course of the disease after first treatment in 14 patients (36%).

Patients had a median number of 2 (range, 1–6) prior lines of therapy prior to alloHCT. Thirteen patients (33%) with *TP53*_mut/del_ diagnosis had received only one line of treatment for remission induction prior to transplantation. Ten of these patients had received alemtuzumab for remission induction.

Altogether 16 patients (41%) had been exposed to purine-analogues (PA), of whom 44% had PA-refractory CLL or experienced relapse within 2 years after PA-containing chemotherapy. Thirty patients (77%) had received alemtuzumab for remission induction prior to transplantation. Alemtuzumab was the last line of treatment in 29 of these 30 patients (97%). At the time of alloHCT, 8 patients (21%) were in CR and 31 patients (79%) were in PR. The majority of patients (62%) received reduced-intensity conditioning. Eleven patients (28%) had HLA-identical sibling donors and 28 patients (82%) had HLA-compatible unrelated donors, including 5 donors with a single mismatch at HLA-A, HLA-B, HLA-C, or HLA-DRB1. Further patient and transplant characteristics are provided in Table S1.

All patients engrafted, and no secondary graft failure was reported. The cumulative incidence of acute GVHD grades II–IV at 100 days was 36% (95% CI, 21–51%). The cumulative incidence of limited or extensive chronic GVHD at 1 year after alloHCT was 63% (95% CI, 48–79%).

Eleven out of 29 patients (38%) with PR prior to transplantation achieved a CR as best response. In the subset of 22 patients who were already MRD-negative at alloHCT or became MRD-negative after alloHCT, 8 relapses were observed and 5 patients died without relapse. The 5-year RFS from first MRD-negative test was 35% (95% CI, 13–58%).

At last follow up, 19 patients were alive with a median follow-up time of 6 years (range, 1–8 years). Eleven patients were alive and MRD-negative at last assessment. One-year RFS was 62% (95% CI, 46–77%). Altogether 17 patients relapsed after HCT. Eleven patients died without relapse after alloHCT. Causes of death were GVHD-related with or without concomitant infection in 7 patients. Estimates for survival, RFS, NRM and relapse incidence and the impact of risk factors are provided in Table 1 and Fig. S1.

**Table 1:** Univariable analysis of overall-free and relapse-free survival, relapse incidence and non-relapse mortality.

Variables	*N*	5-yr OS (95% CI)	*p*-value	5-yr RFS (95% CI)	*p*-value	5-yr CIR (95% CI)	*p*-value	5-yr NRM (95% CI)	*p*-value
Total cohort	39	49 (35–68)		29 (17–48)		42 (26–58)		29 (14–44)	
*Patient age at HCT*									
<60 years	26	46 (29–72)	0.85	25 (12–50)	0.34	51 (31–72)	0.10	24 (6–42)	0.58
≥60 years	13	54 (33–89)		38 (19–76)		23 (0–48)		38 (11–66)	
*HCT-CI, score*									
<3	28	54 (38–77)	0.11	31 (17–54)	0.47	44 (24–63)	0.94	26 (9–43)	0.28
≥3	11	36 (17–79)		27 (10–72)		36 (6–67)		36 (5–68)	
*Detection of deletion(17p)/TP53*_*mut*_									
During Course CLL	14	38 (18–79)	0.84	18 (5–60)	0.32	52 (22–81)	0.26	30 (3–58)	0.84
At diagnosis	25	54 (38–78)		36 (21–61)		36 (17–55)		28 (10–46)	
*Disease stage*									
First remission	13	69 (48–99)	0.12	54 (33–89)	0.04	23 (0–47)	0.050	23 (0–47)	0.40
Advanced stage	26	39 (23–64)		18 (8–42)		51 (30–71)		31 (12–50)	
*Remission status before conditioning*									
Complete remission	8	47 (21–100)	0.92	33 (12–96)	0.51	42 (0–84)	0.56	25 (0–57)	0.76
Partial remission	31	49 (34–71)		28 (15-50)		42 (24–60)		30 (13–48)	
*Alemtuzumab last line prior to HCT*									
No	10	34 (14–85)	0.11	20 (6–69)	0.02	40 (7–73)	0.17	40 (6–74)	0.06
Yes	29	53 (37–76)		32 (18–56)		43 (23–62)		26 (8–44)	
*Donor type*									
HLA-identical sibling	11	36 (17–79)	0.29	27 (10–72)	0.46	45 (13–78)	0.50	27 (0–56)	0.73
Alternative donor	28	53 (37–77)		30 (16–54)		40 (21–59)		30 (12–48)	

*p*-values are based on the log-rank test (OS and PFS) and the Gray test (CIR and NRM), they compare the outcomes of the groups during the whole follow-up.

*OS* overall survival, *RFS* relapse-free survival, *RI* relapse incidence, *NRM* non-relapse mortality, *CI* confidence interval, *HCT* hematopoietic stem cell transplantation, *HCT-CI* hematopoietic cell transplantation-comorbidity index

Patients who had experienced acute GVHD grades II–IV or chronic GVHD had a significantly lower risk of relapse, indicated by a hazard ratio of 0.2 (95% CI, 0.05–0.7, *p* = 0.01) for GVHD modelled as a time-dependent covariate in an extended Cox regression model. Among 11 patients without relapse after transplantation, nine patients (81%) had experienced chronic GVHD which had resolved completely in 5 patients (56%) at last follow-up.

Results for the whole cohort were mixed but showed significant differences for two major subgroups (see Fig. S2). Patients who were referred for alloHCT at advanced disease stages of *TP53*_mut/del_ CLL had disappointing results. Their 5-year incidence of relapse was 51% (95% CI, 30–71%). Most of these patients had acquired *TP53*_mut/del_ as a secondary event after one or more lines of chemoimmunotherapy. Good outcomes were observed among 13 patients who received alloHCT in first remission whose RFS at 5 years was 54% (95% CI, 33–89%) owing to a much lower risk of relapse. Notably, the best subgroup consisted of 10 patients who had received alemtuzumab with or without high-dose corticosteroids for remission induction and proceeded to alloHCT in first remission.

Although the sequence of alemtuzumab-based remission induction and alloHCT in first remission turned out to be most promising in this study, this treatment approach is outdated in the light of today’s treatment options for patients with *TP53*_mut/del_ CLL. For example, in a phase II trial performed by the NIH, the overall response rate (ORR) of patients with *TP53*_mut/del_ CLL on ibrutinib was 96% and treatment naive patients had a 5-year PFS of ~80% [9]. With a fixed-duration treatment with venetoclax and obinutuzumab, mostly elderly patients with *TP53*_mut/del_ CLL and coexisting conditions achieved ORR rates of 85% and 2-year PFS was ~75% [1]. Owing to the more favourable risk-benefit ratio of both oral pathway-inhibitors compared to alloHCT, transplantation in first remission of *TP53*_mut/del_ CLL is no longer recommended [10]. Current guidelines suggest that patients with *TP53*_mut/del_ CLL who failed one pathway-inhibitor or who were intolerant to either BTKi or BCL2i should be referred to a transplant centre [10–12]. Our data indicate that remission induction with alemtuzumab prior to alloHCT should not be excluded for patients with relapsed/refractory *TP53*_mut/del_ CLL who lack better options. Finally, in patients with relapsed *TP53*_mut/del_ CLL, who failed on pathway-inhibitor treatment and have no access to new targeted drugs or CAR-T cells, conventional chemotherapy should be avoided and alloHCT should be considered.

## Supplementary information

Supplementary information
