# Correction to: Allogeneic hematopoietic cell transplantation for patients with *TP53* mutant or deleted chronic lymphocytic leukemia: results of a prospective observational study

**DOI:** 10.1038/s41409-021-01524-2

**Published:** 2021-11-12

**Authors:** Johannes Schetelig, Jennifer Hoek, Stephan Stilgenbauer, Jan Moritz Middeke, Niels Smedegaard Andersen, Christopher P. Fox, Stig Lenhoff, Liisa Volin, Avichai Shimoni, Wilfried Schroyens, Michel van Gelder, Donald Bunjes, Anja van Biezen, Henning Baldauf, Liesbeth C. de Wreede, Olivier Tournilhac, Nicolaus Kröger, Ibrahim Yakoub-Agha, Peter Dreger

**Affiliations:** 1 University Hospital, Dresden, Germany; 2 DKMS Clinical Trials Office, Dresden, Germany; 3 European Society for Blood and Marrow Transplantation (EBMT), Leiden, Netherlands; 4 European Research Initiative on Chronic Lymphocytic Leukemia (ERIC), Barcelona, Spain; 5 EBMT Data Office, Leiden, The Netherlands; 6 Ulm University, Ulm, Germany; 7 Rigshospitalet, Copenhagen, Denmark; 8 Nottingham University Hospitals NHS Trust, Nottingham, UK; 9 University Hospital Lund, Lund, Sweden; 10 Helsinki University Central Hospital, Helsinki, Finland; 11 Chaim Sheba Medical Center, Tel-Hashomer, Israel; 12 Antwerp University Hospital, Edegem, Belgium; 13 University Medical Center Maastricht, Maastricht, The Netherlands; 14 Medical Statistics, Department of Biomedical Data Sciences, Leiden University Medical Center, Leiden, The Netherlands; 15 Service Therapie Cellulaire & Hematologie Clinique, Centre Hospitalier Universitaire, Clermont Ferrand, France; 16 University Hamburg Eppendorf, Hamburg, Germany; 17 Centre Hospitalier Universitaire de Lille, LIRIS, INSERM U995, Université de Lille, Lille, France; 18 Department of Medicine V, University of Heidelberg, Heidelberg, Germany

Correction to: *Bone Marrow Transplantation* (2021) 56:692–695; 10.1038/s41409-020-01013-y, published online 16 August 2020

The Allogeneic hematopoietic cell transplantation for patients with TP53 mutant or deleted chronic lymphocytic leukemia: Results of a prospective observational study, written by Johannes Schetelig JJennifer Hoek, Stephan Stilgenbauer, Jan Moritz Middeke, Niels Smedegaard Andersen,Christopher P. Fox, Stig Lenhoff, Liisa Volin, Avichai Shimoni, Wilfried Schroyens, Michel van Gelder, Donald Bunjes, Anja van Biezen, Henning Baldauf, Liesbeth C. de Wreede, Olivier Tournilhac, Nicolaus Kröger, Ibrahim Yakoub-Agha, Peter Dreger was originally published Online First without Open Access. After publication in volume 56, issue 3, page 692-695 the author decided to opt for Open Choice and to make the article an Open Access publication. Therefore, the copyright of the article has been changed to © The Author(s) 2020 and the article is forthwith distributed under the terms of the Creative Commons Attribution 4.0 International License, which permits use, sharing, adaptation, distribution and reproduction in any medium or format, as long as you give appropriate credit to the original author(s) and the source, provide a link to the Creative Commons licence, and indicate if changes were made. The images or other third party material in this article are included in the article’s Creative Commons licence, unless indicated otherwise in a credit line to the material. If material is not included in the article’s Creative Commons licence and your intended use is not permitted by statutory regulation or exceeds the permitted use, you will need to obtain permission directly from the copyright holder. To view a copy of this licence, visit http://creativecommons.org/licenses/by/4.0. Open access funding enabled and organized by Projekt DEAL.

