# Supplementary material for: Allogeneic hematopoietic cell transplantation for patients with TP53 mutant or deleted chronic lymphocytic leukemia: Results of a prospective observational study
Source: Bone Marrow Transplant. 2020 Aug 16;56(3):692–5. doi: 10.1038/s41409-020-01013-y (PMC8589674; doi:10.1038/s41409-020-01013-y)
Supplement: Supplementary file 1 — Supplementary information [file 41409_2020_1013_MOESM1_ESM.doc]

Allogeneic hematopoietic cell transplantation for Patients with *TP53* mutant or deleted Chronic Lymphocytic Leukemia: Results of a prospective observational study

Running head: AlloHCT for *TP53*_mut/del_ CLL

First author: Johannes Schetelig

Supplement

TABLE OF CONTENTS

[Patients and Methods 3](#__RefHeading___Toc43396075)

[Study Design & Patient Eligibility 3](#__RefHeading___Toc43396076)

[Data Management 3](#__RefHeading___Toc43396077)

[Statistical Analysis 4](#__RefHeading___Toc43396078)

[Results 5](#__RefHeading___Toc43396079)

[Patient Characteristics 5](#__RefHeading___Toc43396080)

[Transplant Procedure 6](#__RefHeading___Toc43396081)

[Engraftment and GVHD 6](#__RefHeading___Toc43396082)

[Response 7](#__RefHeading___Toc43396083)

[Overall Survival, Relapse-free Survival, Relapse and Non-Relapse Mortality 7](#__RefHeading___Toc43396084)

[Univariable Comparisons 8](#__RefHeading___Toc43396085)

[Impact of GVHD 8](#__RefHeading___Toc43396086)

[Participating Centers not acknowledged by Coauthorship 9](#__RefHeading___Toc43396087)

[Supplementary Table S1. Patient characteristics 10](#__RefHeading___Toc43396088)

[Supplementary Figure S1. 11](#__RefHeading___Toc43396089)

[Supplementary Figure S2. Outcomes by Disease Stage 13](#__RefHeading___Toc43396090)

# Patients and Methods

## Study Design & Patient Eligibility

The study was conducted as a registry-based prospective observational study by the Chronic Malignancies Working Party of EBMT. Transplant Centres with an active CLL transplant program, which included regular measurements of minimal residual disease were requested to register all eligible patients with this study at the time of alloHCT. Eligibility criteria were as follows: Patients had to be scheduled to receive HLA-compatible related or unrelated alloHCT for 17p-/*TP53*-mutated CLL. Patients had to be in first or second complete or partial remission at the time of alloHCT. Exclusion criteria were more than one mismatch at the HLA loci A, B, C or DRB1, patient age ≥70 years, *ex vivo* T-cell depletion of the graft or intended *in vivo* T-cell depletion with alemtuzumab. All patients signed informed consent. The trial was conducted in full compliance with the declaration of Helsinki and was registered with clinicaltrials.gov (NCT01675102).

## Data Management

For registered patients, baseline information, characteristics of the transplant procedure and outcome data were collected on standard Minimal Essential Data A and B forms of EBMT. Dose-intensity of the conditioning regimen was classified according to consensus working definitions of EBMT and CIBMTR[17]. Patients who achieved a complete remission were tested for minimal residual disease (MRD) in peripheral blood and bone marrow. Testing for MRD was done locally using flow cytometry or molecular analyses.

## Statistical Analysis

The primary aim of the study was to analyse relapse-free survival (RFS) at one year after alloHCT. Events for RFS were clinical relapse and death. Immune manipulations such as the taper of immunosuppressive drugs, the administration of donor lymphocyte infusions or the administration of rituximab were not considered as events for RFS. Non-relapse mortality (NRM) was defined as death without preceding clinical relapse after alloHCT.

The required sample size was determined such that in the primary efficacy analysis the null hypothesis could be tested that the RFS-probability was 50% or less at one year after alloHCT. Based on a single stage phase II design according to Fleming A'Hern (1982) with a power of 80%, a one-sided type one error of 5%, and a drop-out rate of 10%, 41 patients were included[18].

Curves for RFS and overall survival (OS) were calculated using the Kaplan-Meier method and compared between groups by log rank tests. Cumulative incidences of relapse and NRM were calculated using competing risks methods and between-group comparisons were performed with the Gray test[19]. The cumulative incidences of acute GVHD and chronic GVHD were also calculated in a competing risks framework, considering death before acute/chronic GVHD, respectively, as competing event. The impact of the occurrence of GVHD (acute GVHD grades II to IV or any grade of chronic GVHD) on the incidence of relapse was tested in a time-dependent cause-specific Cox-regression model. All point estimates for time-to-event endpoints are reported together with 95%-confidence intervals. Data were analyzed as of August 31, 2019.

# Results

## Patient Characteristics

A total of 41 patients were enrolled between June 2010 and September 2012. One patient with Richter’s Syndrome and one patient aged >70 years at alloHCT did not meet the eligibility criteria, and were excluded from the study after review of the data. The final analysis set therefore included data from 39 patients.

Patient characteristics are shown in Table 1. Twenty-eight male and 11 female patients were enrolled at 11 centers. The median age was 59 years (range, 28 to 69 years). The Karnofsky Performance Score was ≥90% for 85% of patients and 80% for the remaining patients. The median Hematopoietic Cell Transplantation - Comorbidity Index was 0 (range, 0 to 9) but 28% of patients had a score ≥3.

The median interval between diagnosis of CLL and alloHCT was 35 months (range, 4 to 229 months), and the median time from first treatment of CLL to alloHCT was 12 months (range, 2 – 227 months). Thirty-five patients (90%) had a deletion 17p confirmed by FISH with or without a *TP53*-mutation, while 4 patients (10%) had a *TP53*-mutation only. The deletion (17p)/*TP53*-mutation was diagnosed during the course of the disease after first treatment in 14 patients (36%).

Patients had been treated with a median number of 2 (range, 1 to 6) prior lines of therapy prior to alloHCT. Thirteen patients (33%) with *TP53*_del/mut_ diagnosis had received just one line of treatment for remission induction prior to transplantation. Ten of these patients who were referred for transplantation in first remission had received alemtuzumab for remission induction. Sixteen patients (41%) had been exposed to purine-analogues, of whom 44% had purine-analogue refractory CLL or experienced relapse within 2 years after purine-analogue containing chemotherapy. In total, 30 patients (77%) had received alemtuzumab for remission induction prior to transplantation, and alemtuzumab was the last line of treatment in almost all of these patients (97%). At the time of alloHCT, 8 patients (21%) were in complete remission from CLL, and the remaining patients (79%) were in partial remission.

## Transplant Procedure

Twenty-four patients (62%) received reduced-intensity conditioning based on combinations of fludarabine and either busulfan, melphalan, or cyclophosphamide. Twelve patients (31%) received non-myeloablative conditioning based on 2 Gray total body irradiation (TBI) and three patients (8%) received myeloablative conditioning. Eleven patients (28%) received hematopoietic stem cells from HLA-identical siblings, while 28 patients (82%) had HLA-compatible unrelated donors, including 5 patients whose donors had a single mismatch at HLA-A, -B, -C, or HLA-DRB1. G-CSF-mobilised peripheral blood stem cells were used as graft source in 36 patients (93%), two patients received bone marrow and one patient cord blood. GVHD prophylaxis was based on ciclosporin with methotrexate or mycophenolate mofetil in 36 patients (92%). Anti-thymocyte globulin (ATG) was part of the conditioning regimen in 13 patients (33%).

## Engraftment and GVHD

All patients engrafted, and no secondary graft failure was reported. The cumulative incidence of acute GVHD grades II-IV at 100 days was 36% (95%-CI, 21% to 51%). The cumulative incidence of limited or extensive chronic GVHD at 1 year after alloHCT was 63% (95%-CI, 48% to 79%).

## Response

Eleven out of 29 patients (38%) with PR prior to transplantation achieved a complete remission as best response. In order to evaluate the impact of MRD-negativity after HCT we performed a subgroup analysis of patients who were MRD-negative prior to the start of the conditioning regimen or achieved MRD-negativity any time after HCT. In this subset of 22 patients, 8 relapses were observed and 5 patients died without relapse. Five-year relapse-free survival from first MRD-negative test was 35% (95%-CI, 13% to 58%). All eleven patients, who were alive and in remission at last follow up, were MRD-negative at last assessment.

## Overall Survival, Relapse-free Survival, Relapse and Non-Relapse Mortality

At last follow up, 19 patients were alive with a median follow-up time of 6 years (range, 1 to 8 years). One-year RFS was 62% (95%-CI, 46% to 77%). The probability of OS and RFS at 5 years was 49% (95%-CI, 35% to 68%) and 29% (95%-CI, 17% to 48%), respectively. Altogether 17 patients had a relapse after HCT. Relapse occurred within the first year after HCT in 7 patients. The latest relapse observed up to the time of this analysis occurred 65 months after HCT. The cumulative incidence of relapse at 5 years was 42% (95%-CI, 26% to 58%).

Eleven patients died without relapse after alloHCT. The cumulative incidence of NRM at 5 years was 29% (95%-CI, 14% to 44%). Causes of death were assessed as HCT-related in 10 patients, with 7 fatalities related to GVHD with or without concomitant infection. An intracranial haemorrhage 2 ½ years after HCT in a 69 year old patient in remission was reported as unrelated to HCT.

## Univariable Comparisons

In exploratory univariable analyses, age, HCT-CI, primary versus secondary TP53-lesions, remission status, alemtuzumab treatment for remission induction prior to HCT, and donor type did not have a significant impact on OS, RFS, NRM and CIR (see Table 2). In contrast, disease stage had a significant impact on RFS. Patients who were transplanted in first remission had a significantly better 5-year RFS (54% versus 18%, log-rank test p=0.04) compared to patients with more advanced disease. One-year and two-year RFS were 58% (95%CI, 40% to 76%) and 42% (95%CI, 24% to 60%) for patients with advanced *TP53*_del/mut_ CLL.

Notably, ten of the patients transplanted in first remission (77%) had received only alemtuzumab with or without high-dose corticosteroids for remission induction prior to alloHCT. The 5-year OS and RFS for these 10 patients was 80% (95%-CI, 55%% to 100%) and 60% (95%-CI, 29% to 91%). The main reason for treatment failure among patients with more advanced CLL was relapse which amounted to a cumulative incidence of 51% (95%CI, 30% to 51%) at 5 years.

## Impact of GVHD

Patients who had experienced acute GVHD grades II to IV or chronic GVHD had a significantly lower risk of relapse, indicated by a hazard ratio of 0.2 (95% CI, 0.05 to 0.7, p=0.01) for GVHD modelled as a time-dependent covariate in an extended Cox regression model. Among the 11 patients without relapse after transplantation, nine patients (81%) had experienced chronic GVHD at any time after transplantation. Chronic GVHD resolved in 5 out of 9 patients (56%) who were free of chronic GVHD at last follow-up.

# Participating Centers not acknowledged by Coauthorship

Belgium Cliniques Universitaires St. Luc, Brussels (A. Ferrant)

Czech Republic Institute of Hematology and Blood Transfusion, Prague (A. Vitek)

Finland Helsinki University Central Hospital, Helsinki (R. Niittyvuopio)

Central Hospital, Turku (M. Itala)

France Hopital La Miletrie, Poitiers (F. Guilhot)

Germany Charité, University Hospital Benjamin Franklin, Berlin (L. Uharek)

University Hospital, Göttingen (B. Glass)

AK St. Georg, Hamburg (N. Schmitz)
 University Hospital, Hannover (A. Ganser)

Dept. Medicine II, University Hospital; Kiel (M. Kneba)

University Hospital, Münster (M. Stelljes)

Deutsche Klinik für Diagnostik, Wiesbaden (R. Schwerdtfeger)

Italy Ospedale Maggiore di Milano, Milano (F. Onida)

Istituto Nazionale dei Tumori (P. Corradini)

Spain Hospital St. Creu I St. Pau, Barcelona (R. Martino)

Clinica Puerta de Hierro, Madrid (J.A. García-Marco)

Hospital Clínico, Salamanca (D. Caballero)

Hospital Universitario La Fe, Valencia (M.A. Sanz)

## Supplementary Table S1. Patient characteristics

|  | Numbers of Patients (%)  Total, N=39 |
| --- | --- |
| Median age at HCT [years] (range)  Patients older than 60 years | 59 (28 to 69)  13 (33) |
| Female  Male | 11 (28)  28 (72) |
| Karnofsky index at HCT  100%  90%  80% | 11 (28)  22 (56)  6 (15) |
| Median HCT-CI (range)  Patients with HCT-CI ≥3 | 0 (0 to 9)  11 (28) |
| Previous Lines of Therapies, median (range)  containing, N of patients  Fludarabine   Rituximab  Alemtuzumab | 2 (1 to 6)  16 (41) 18 (46)  30 (77) |
| Remission Status at HCT  Complete Remission  Partial Remission | 8 (21)  31 (79) |
| Donor type  HLA-identical sibling  Matched unrelated donor  Partially matched unrelated donor | 11 (28)  23 (59)  5 (13) |
| CMV constellation  Donor and recipient CMV neg.  Donor or recipient CMV pos. | 13 (33)  26 (67) |
| Sex Constellation  Female patient – female donor  Female patient – male donor  Male patient – female donor  Male patient – male donor | 6 (15)  5 (13)  8 (21)  20 (51) |
| Conditioning regimen  Non-myeloablative based on 2 Gray TBI  Reduced intensity  High-dose therapy | 12 (31)  24 (62)  3 (8) |
| Stem cell source  Peripheral Blood Stem Cells  Bone marrow  Cord Blood | 36 (92)  2 (5)  1 (3) |
| GVHD prophylaxis  CSA plus MMF  CSA plus MTX  Other  Administration of ATG | 13 (33)  23 (59)  3 (8)  13 (33) |

Legend: N, number; HCT, hematopoietic cell transplantation; HCT-CI, Hematopoietic Cell Transplantation – Comorbidity Index; CMV, cytomegalovirus; GVHD, graft-versus-host disease; TBI, total body irradiation ; CSA, cyclosporine A; MTX, methotrexate; MMF, mycophenolate mofetil; PBSC, peripheral blood stem cells; ATG, anti-thymocyte globulin

## Supplementary Figure S1.

Kaplan Meier Plots with 95%-Confidence Intervals for Overall (Panel S1A) and Relapse-free Survival (Panel S1B), Ccumulative Incidences of Relapse and Non-Relapse Mortality (Panel S1C)

| Panel S1A  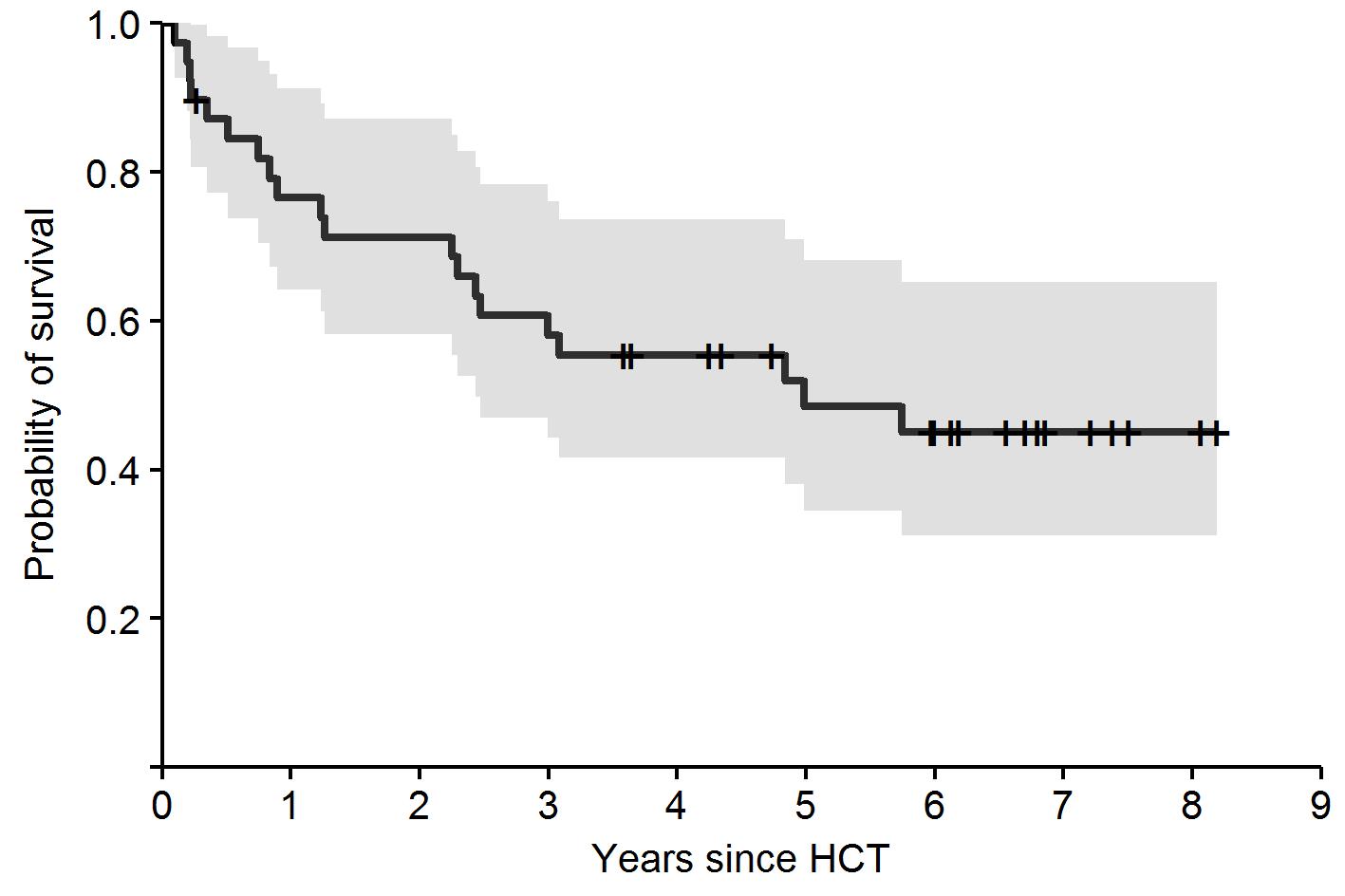 |
| --- |
| Panel S1B  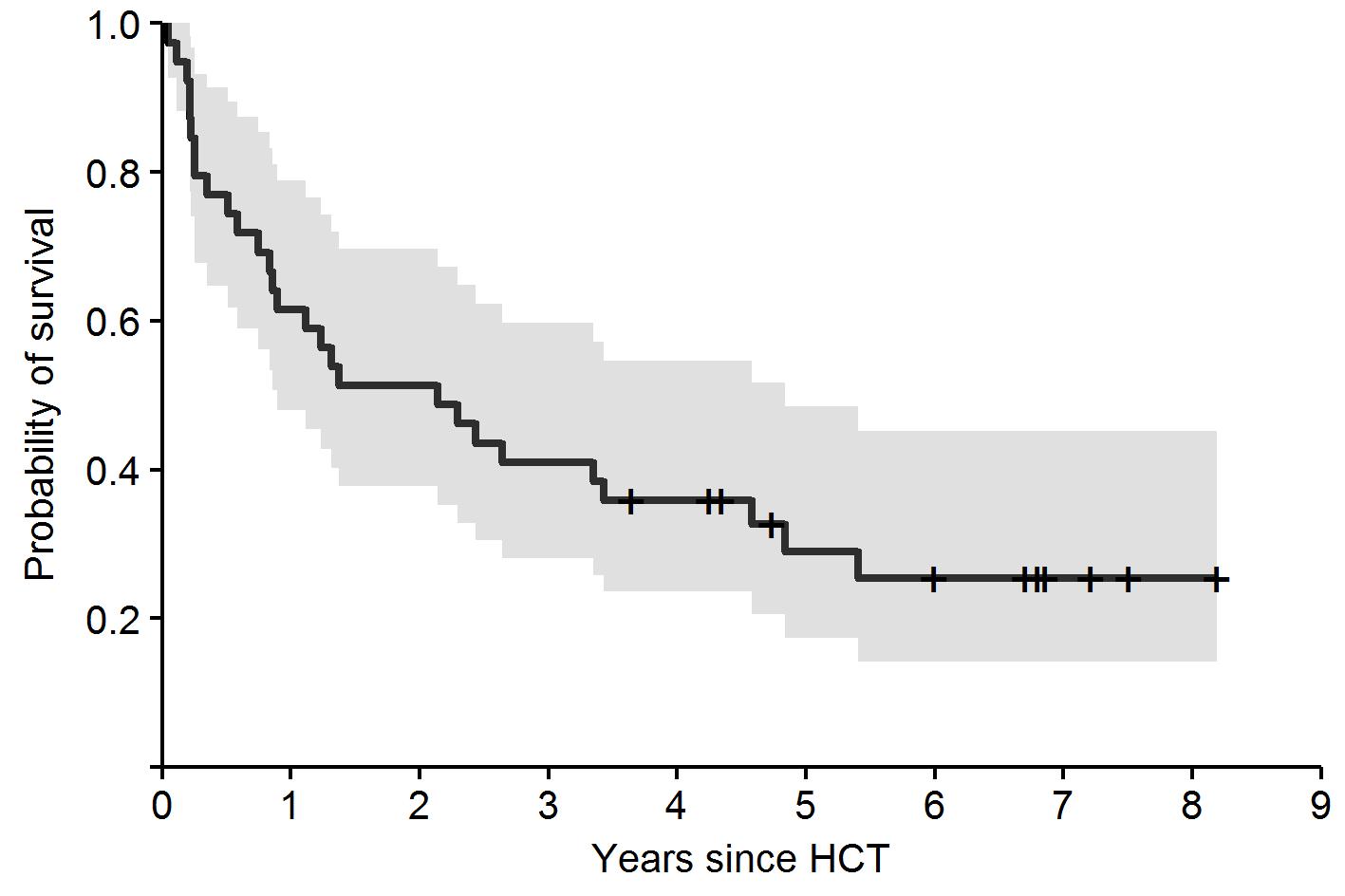 |

| Panel S1C  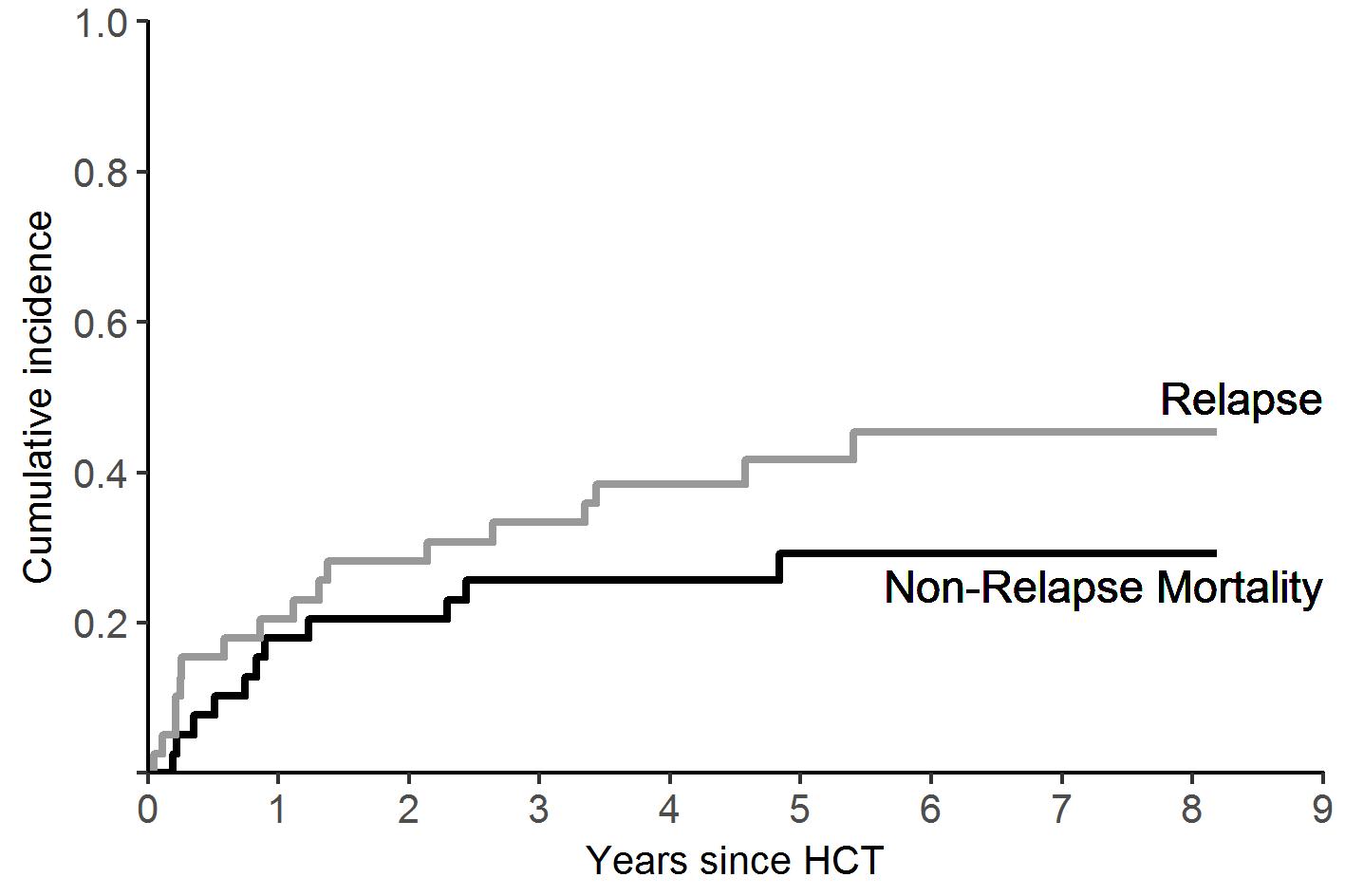 |
| --- |

## Supplementary Figure S2. Outcomes by Disease Stage

Relapse-free survival (Panel S2A) of patients who received HCT in first remission versus advanced disease stages. Cumulative incidences of relapse and non-relapse mortality in first remission (Panel S2B) and more advanced disease stages (Panel S2C).

| Panel S2A  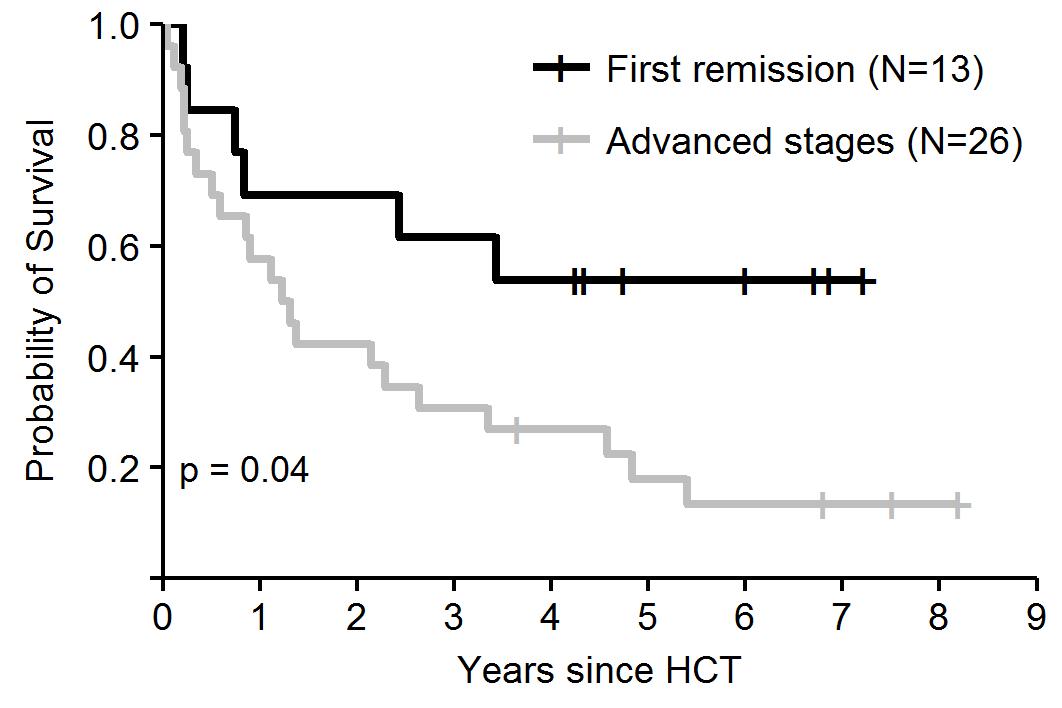 |
| --- |
| Panel S2B  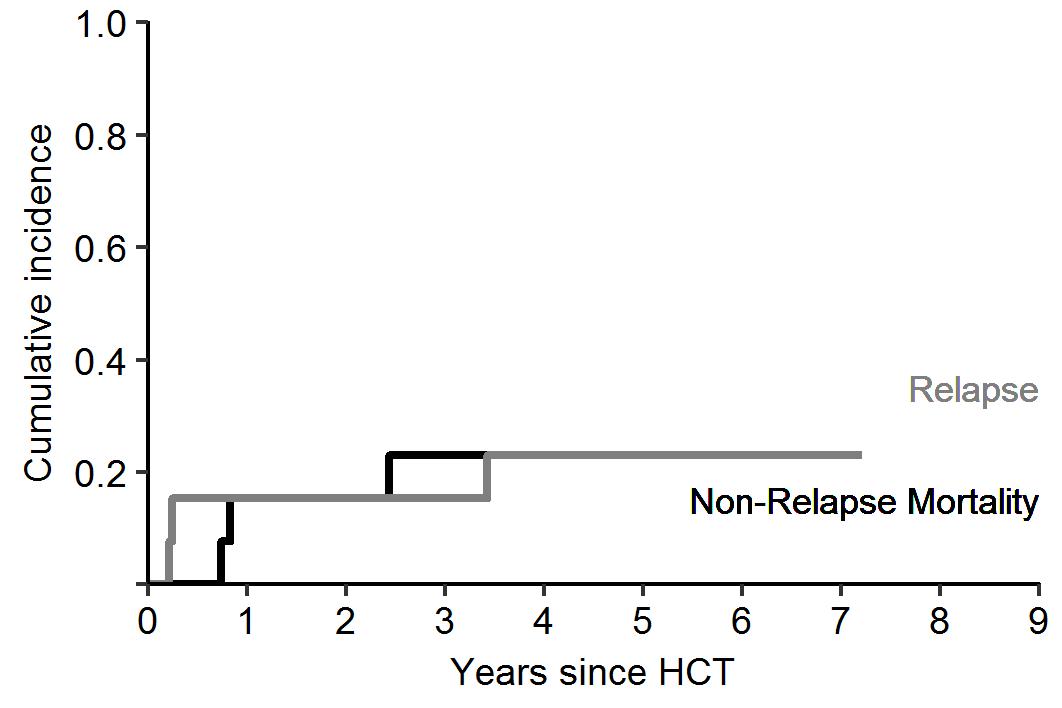 |

| Panel S2C  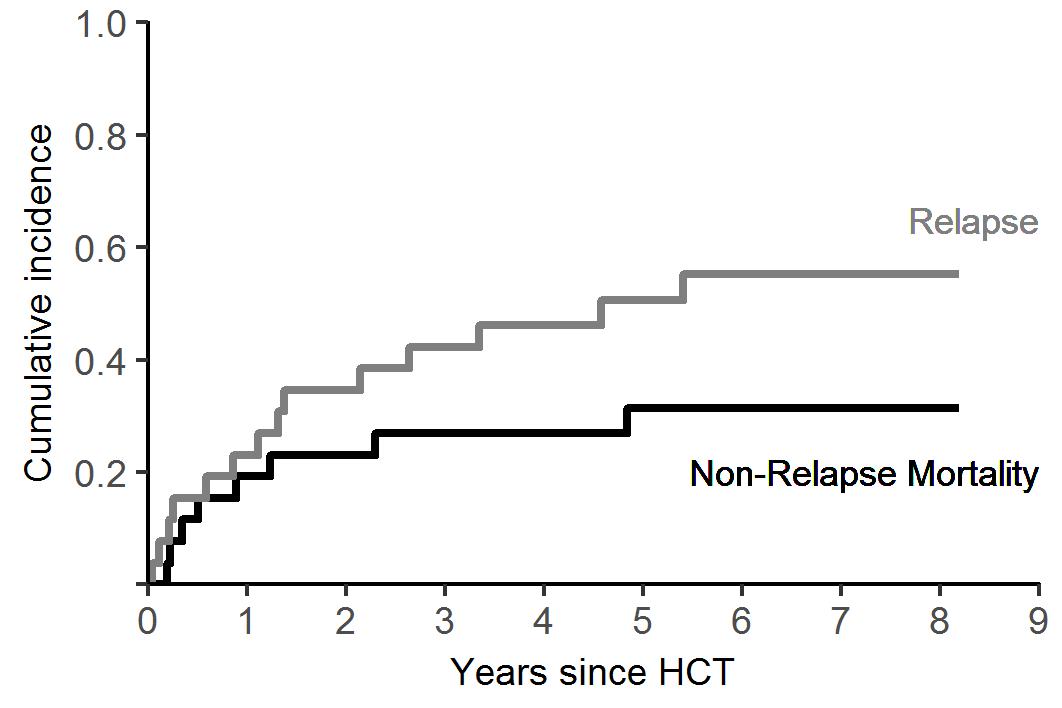 |
| --- |
